# Fibrosis markers as prognostic markers of decline in kidney function in patients with neuroendocrine neoplasms undergoing peptide receptor radionuclide therapy

**DOI:** 10.3389/fendo.2025.1495369

**Published:** 2025-06-27

**Authors:** Tobias Stemann Lau, Lars Bossen, Daniel Guldager Kring Rasmussen, Federica Genovese, Morten Karsdal, Anne Kirstine Arveschoug, Henning Grønbæk, Gitte Dam

**Affiliations:** ^1^ Department of Hepatology and Gastroenterology, Aarhus University Hospital, Aarhus, Denmark; ^2^ European Neuroendocrine Tumor Society (ENETS) Center of Excellence, Aarhus University Hospital, Aarhus, Denmark; ^3^ Department of Clinical Medicine, Aarhus University, Aarhus, Denmark; ^4^ Nordic Bioscience A/S, Herlev, Denmark; ^5^ Department of Nuclear Medicine and PET-Centre, Aarhus University, Aarhus, Denmark

**Keywords:** PRRT, fibrosis markers, biomarkers, neuroendocrine neoplasms, neuroendocrine tumors, kidney fibrosis

## Abstract

**Introduction:**

Decline in kidney function due to renal fibrosis is a potential side effect in patients with neuroendocrine neoplasm (NEN) undergoing Peptide Receptor Radionuclide Therapy (PRRT). We aimed to investigate the potential of circulating fibrosis markers reflecting formation and degradation of collagens in predicting decline in kidney function in NEN-patients undergoing PRRT.

**Material and methods:**

We included NEN-patients referred for PRRT treatment. We measured two biomarkers of type III and VI collagen formation, reflecting fibrogenesis (PRO-C3 and PRO-C6), and a degradation biomarker of type III collagen, reflecting fibrolysis (C3M) in serum and urine before initiation of PRRT and after each treatment. A kidney function test was performed before initiation of PRRT and three months after end of treatment (EOT) and when possible 6, 12, 18, and 24 months after EOT. We performed a linear mixed model to evaluate differences in the levels of fibrosis markers between patients who declined in kidney function *vs* patients who did not.

**Results:**

Fourteen patients (57% men and median age 67 years (IQR: 61-75)), completed PRRT treatment with at least one kidney function test following EOT. Median time from EOT to last kidney function test was 12 months (IQR: 12-21). Six patients (43%) experienced a more than 25% decline in kidney function from baseline to last kidney function test. For urinary (u) C3M, the overall linear mixed model was marginally significant (p = 0.078). Specifically, after the first treatment (74 ng/mg (95% CI: 49-113) *vs* 135 ng/mg (95% CI: 93-194)) and three months after EOT (56 ng/mg (95% CI: 37-86) *vs* 118 (95% CI: 81-173)), levels of uC3M were significantly lower in patients who subsequently had decline in kidney function.

**Conclusion:**

At specific time points, levels of uC3M significantly differed in patients who subsequently declined in kidney function. From these exploratory results, we believe that uC3M holds the potential as a prognostic biomarker, and we suggest that this should be further investigated in larger studies to draw firm conclusions about the usefulness.

## 1 Introduction

Neuroendocrine neoplasms (NENs) are tumors arising from neuroendocrine cells distributed throughout the body, most often in the gastroenteropancreatic system or the lungs. Depending on the production of hormones and other vasoactive substances, patients present with different symptoms and complications. The prognosis differs significantly depending on differentiation and grade (1, 2).

Fibrosis development is a common complication to NENs and affects both morbidity and mortality (3) and occurs both locally adjacent to the tumor and at distant sites. Overall, fibrosis development is either tumor-dependent due to hormone production or treatment induced. Even though not fully understood, serotonin is thought to be the main driver of tumor-dependent fibrosis which encompasses mesenteric desmoplasia and carcinoid heart disease (4, 5).

Peptide receptor radionuclide therapy (PRRT) is a well-established treatment for patients with disseminated NENs and can lead to irreversible renal impairment and chronic kidney disease (CKD) due to treatment-induced fibrosis (6–9). In the presence of CKD, further treatment options are limited, and the mortality is increased. No available biomarkers can identify particularly vulnerable patients that are at increased risk of kidney impairment following PRRT treatment. Such biomarkers would be of great interest to tailor and personalize treatment and minimize the risk of future irreversible kidney damage in PRRT treated NEN patients.

CKD is characterized by renal (tubulo-interstitial) fibrosis formation with an imbalanced collagen turnover resulting in excessive collagen deposition in the extracellular matrix (10–14). This is the case regardless of the underlying etiology. During both formation and degradation of collagens, small measurable fragments are released into the circulation. These fragments are measurable when structural changes occur even before any functional manifestations occur. In fact, changes in fibrosis marker levels may happen before irreversible fibrosis is present and before any decline in the glomerular filtration rate has occurred (14) which underlines the huge potential for these markers in detecting fibrosis at very early stages.

In recent years, several markers reflecting formation and degradation of collagens have been investigated as both diagnostic, prognostic, and predictive markers in a wide range of diseases including different cancers and liver-, lung-, and kidney fibrosis. Two of the markers, the collagen type VI formation marker PRO-C6 and the collagen type III degradation marker C3M correlated to kidney function in cross-sectional studies (15–19). Interestingly, they also showed potential in predicting adverse renal outcomes in prospective studies in different cohorts of patients with kidney disease (17, 20–25). In patients with NENs, no prospective studies have investigated fibrosis markers as predictors of a decline in kidney function. In a previous cross-sectional study, we demonstrated an association between kidney function and serum PRO-C6, urine PRO-C6, and urine C3M in NEN-patients who had finished PRRT treatment (15). In the present study, we hypothesize that the fibrosis markers hold potential as prognostic markers. Therefore, we aimed to prospectively investigate the potential of these fibrosis markers in predicting decline in kidney function in NEN-patients undergoing PRRT.

## 2 Methods

### 2.1 Study design and patients

This study was an observational, prospective cohort study conducted at Aarhus University Hospital, Denmark at the Department of Hepatology and Gastroenterology, ENETS Center of Excellence in collaboration with Department of Nuclear Medicine and PET Centre.

Eligible patients were patients diagnosed with NEN (all primary localizations) prior to PRRT treatment. Exclusion criteria were age under 18 years and other cancers than NEN. Patients were recruited from the Department of Hepatology and Gastroenterology, ENETS Center of Excellence at Aarhus University Hospital, Denmark.

### 2.2 Procedures

#### 2.2.1 PRRT

All included patients were treated with PRRT on a clinical indication and by standard protocols and renal protection protocols following international guidelines at the Department of Nuclear Medicine and PET Centre at Aarhus University Hospital from 2017 to 2019 (26, 27). Isotopes used were either ^177^Lu or ^90^Y. The interval between two PRRT treatments were between 8 and 14 weeks. Usually, a PRRT treatment cycle consists of four treatments.

#### 2.2.2 Kidney function test

Before the first PRRT treatment, all patients had their baseline kidney function determined by either ^51^Cr-EDTA or ^99^mTc-DTPA plasma clearance at the Department of Nuclear Medicine and PET Centre at Aarhus University Hospital, Denmark. For all patients, a follow up kidney function test was performed three months after the last PRRT treatment, and if possible after 6 months, 12 months, 18 months, and 24 months. This was part of the standard clinical follow up after PRRT. The measured kidney function obtained by ^51^Cr-EDTA or ^99^mTc-DTPA plasma clearance is expressed as ‘Standard-GFR’.

#### 2.2.3 Blood- and urine samples

Baseline blood- and urine samples for measurement of fibrosis markers were drawn before PRRT treatment (visit 1); either the day before or at the same day as the first PRRT. Follow up blood- and urine samples were drawn either the day before or at the same day as the following PRRT treatment. This was repeated before all PRRT treatments (visit 2, 3, and 4). Time between two PRRT treatments were between 8 and 14 weeks. The last follow up blood- and urine samples were drawn three months after the last PRRT treatment (visit 5).

### 2.3 Biochemical analysis

Three different fibrosis markers were measured in both serum and urine. A type III collagen formation biomarker (PRO-C3), a type VI collagen formation biomarker (PRO-C6), and a type III degradation biomarker (C3M). Measurements of the fibrosis markers were carried out as previously described (15). Urine measurements were normalized by urine creatinine and carried out as previously described (15).

### 2.4 Statistics

Patient baseline characteristics are presented as median and interquartile range (IQR) or number and percentage. Patients were divided into two groups according to development in their kidney function; those who experienced a 25% or more decline in kidney function during follow up (i.e., change from baseline test to the last follow up test available) (decline in kidney function), and those who did not (preserved kidney function). Comparisons between those two groups were performed using χ^2^ test or Fisher’s exact test for categorical variables and Mann-Whitney U test for continuous variables.

A linear mixed model with random intercept was used to estimate means of each of the fibrosis markers at all visits and to evaluate overall differences between the two groups for each biomarker. If the overall linear mixed model was statistically significant (or marginally significant) for a specific biomarker, we went on to evaluate differences of the estimated means both within groups and between the two groups using the linear mixed model. All analyses performed in the linear mixed model were performed on log-transformed data and were back-transformed when reported.

Finally, we evaluated the cross-sectional correlations between each of the fibrosis markers and the kidney function at baseline and three months after the last treatment. This was done using the Spearman’s correlation.

## 3 Results

### 3.1 Baseline characteristics

**Figure 1:**
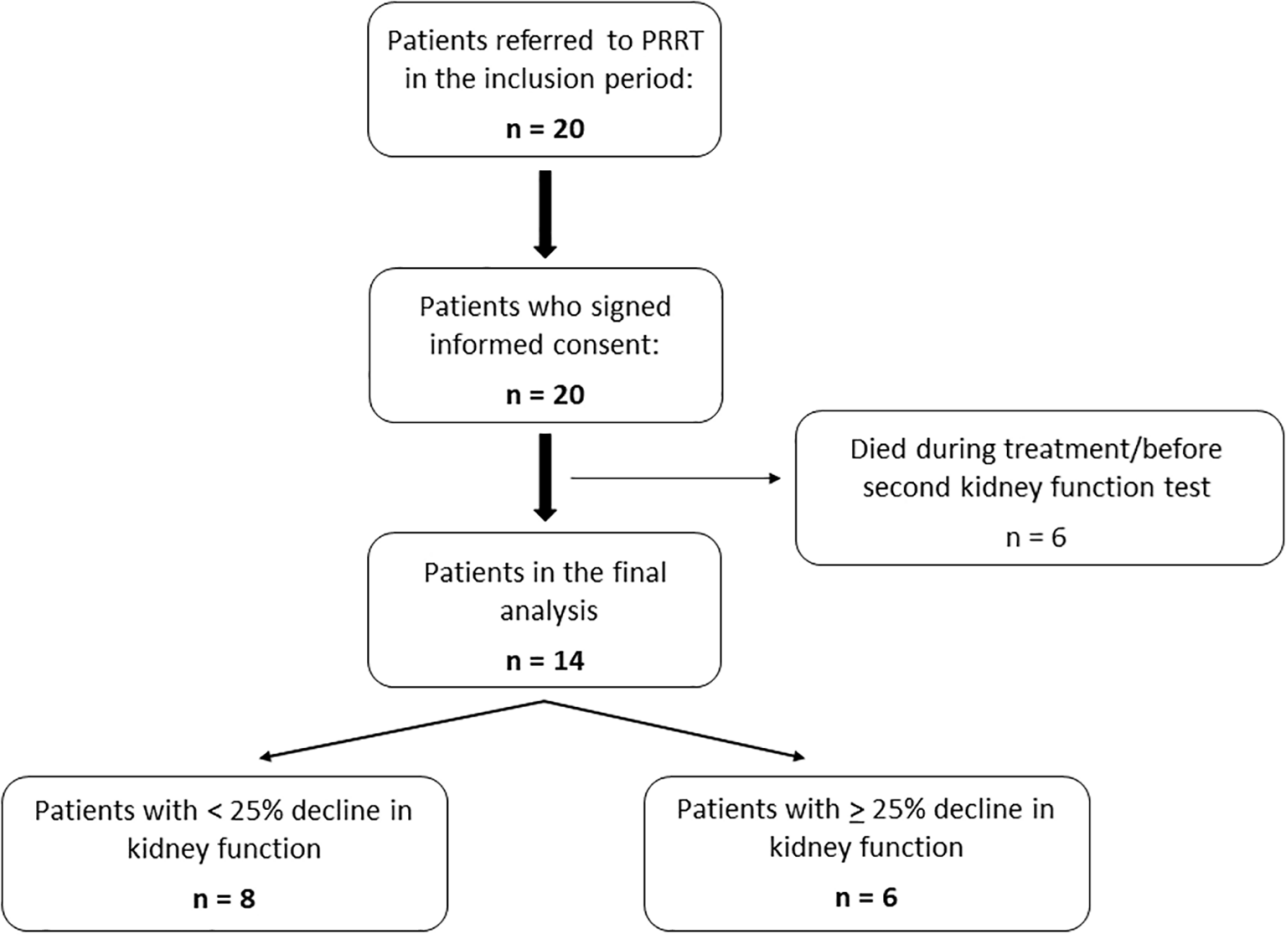
Flow chart of patients included.

A total of 20 patients were referred for PRRT in the recruitment period and all accepted enrollment in the study. Six of these died during treatment before a follow up kidney function test was performed. Thus, 14 patients were included in the final analysis. Patients were divided into two groups; Those who experienced a 25% or more decrease in kidney function during the follow period compared to baseline (decline in kidney function), and those who did not (preserved kidney function) (**Figure 1**).

Eight (57%) of the patients were men, and the median age was 67 years. Most common tumor localization was the small intestine (57%) followed by pancreas (36%) and lungs (7%). There were no major differences in baseline characteristics between the two groups (**Table 1**).

**Table 1:** Baseline characteristics.

	All (n=14)	Preserved kidney function (n=8)	Decline in kidney function (n=6)
Sex			
*Men*	8 (57%)	4 (50%)	4 (67%)
*Women*	6 (43%)	4 (50%)	2 (33%)
Age, years	67 (61-75)	69 (62-79)	67 (61-67)
Weight, kg	74 (61-84)	71 (60-79)	79 (61-90)
BMI	25 (23-28)	23 (23-26)	27 (24-29)
Hypertension	7 (50%)	3 (38%)	4 (67%)
Diabetes mellitus	0 (0%)	0 (0%)	0 (0%)
Previous nephrotoxic treatment*	5 (36%)	3 (38%)	2 (33%)
Previous PRRT	5 (36%)	2 (25%)	3 (50%)
Tumor localization			
*Small intestine*	8 (57%)	3 (38%)	5 (83%)
*Pancreas*	5 (36%)	4 (40%)	1 (17%)
*Lung*	1 (7%)	1 (12%)	0 (0%)
Ki67-index, %	10 (1-15)	13 (9-20)	3 (1-15)
Isotope			
*Lutetium*	8 (57%)	5 (63%)	3 (50%)
*Yttrium*	1 (7%)	0 (0%)	1 (17%)
*Both*	5 (36%)	3 (37%)	2 (33%)
Number of treatments	4 (4-4)	4 (4-4)	4 (4-4)
Chromogranin A, pmol/l	2050 (1030-4430)	2570 (712-4490)	2050 (1620-3240)
P-Creatinine, μmol/l	80 (57-98)	71 (52-83)	97 (78-104)
eGFR, ml/min/1.73m^2^	81 (64-90)	89 (67-90)	67 (64-89)
Standard-GFR, ml/min/1.73m^2^	80 (62-95)	89 (63-103)	71 (55-87)
U-Creatinine, mmol/l	6.5 (2.9-10.5)	7.6 (3.8-11.1)	4.7 (2.6-8.7)

Data are n (%) or median (IQR). *Have previously received Streptozocin/5-Fluorurcil. There were no statistically significant differences between patients with preserved kidney function and patients with decline in kidney function.

### 3.2 Kidney function

Six patients (43%) developed a more than 25% decline in kidney function from their baseline kidney function test to the last kidney function test available. For this group of patients, the baseline median standard-GFR was 71 ml/min/1.73 m^2^ (IQR: 55-87) and the median standard-GFR at the last kidney function test was 44 ml/min/1.73 m^2^ (IQR: 33-50). Three months after EOT, the median standard-GFR was 54 ml/min/1.73 m^2^ (IQR: 50-75). For the group of patients with preserved kidney function, the baseline median was 89 ml/min/1.73 m^2^ (IQR: 63-103) and the end of follow-up median was 87 ml/min/1.73 m^2^ (IQR: 56-98). Three months after EOT the median standard-GFR was 85 ml/min/1.73 m^2^ (IQR: 61-91) (**Table 2**). The median follow-up time from end of PRRT treatment to last kidney function test was 12 months (IQR: 12-21) for all patients. For the group of patients with decline in kidney function, the follow-up time was 16.5 months (IQR: 12-23) and for the group with preserved kidney function, the follow-up time was 12 months (IQR: 9-16).

**Table 2:** Baseline and follow-up kidney function according to group.

Preserved kidney function (n = 8)			Decline in kidney function (n = 6)		
Baseline	3 months after EOT	End of follow-up	Baseline	3 months after EOT	End of follow-up
89 (63 – 103)	85 (61 – 91)	87 (56 – 98)	71 (55 – 87)	54 (50 – 75)	44 (33 – 50)

Data are median (IQR). The kidney function is expressed as measured glomerular filtrations rate (ml/min/1.73m^2^). EOT: end of PRRT treatment. Follow-up kidney function is the last available kidney function test after end of treatment.

The estimated glomerular filtration rates (eGFR) from each visit are shown in **Supplementary Table 3**.

The cumulative PRRT dose administered was 29.9 Giga becquerel (GBq) (21.7 – 29.6) (median (IQR)) for patients with preserved kidney function and 25.8 GBq (20.1 – 29.6) for patients who declined in kidney function.

### 3.3 Fibrosis markers

The linear mixed model was performed for all biomarkers to test for overall differences between the two groups. For uC3M, the overall model revealed marginally significance (p = 0.078), while for the rest of the biomarkers the overall linear mixed model was non-significant (sPRO-C3: p = 0.52, sPRO-C6: p = 0.14, sC3M: p = 0.77, uPRO-C3: p = 0.22, uPRO-C6: p = 0.56).

**Figure 2:**
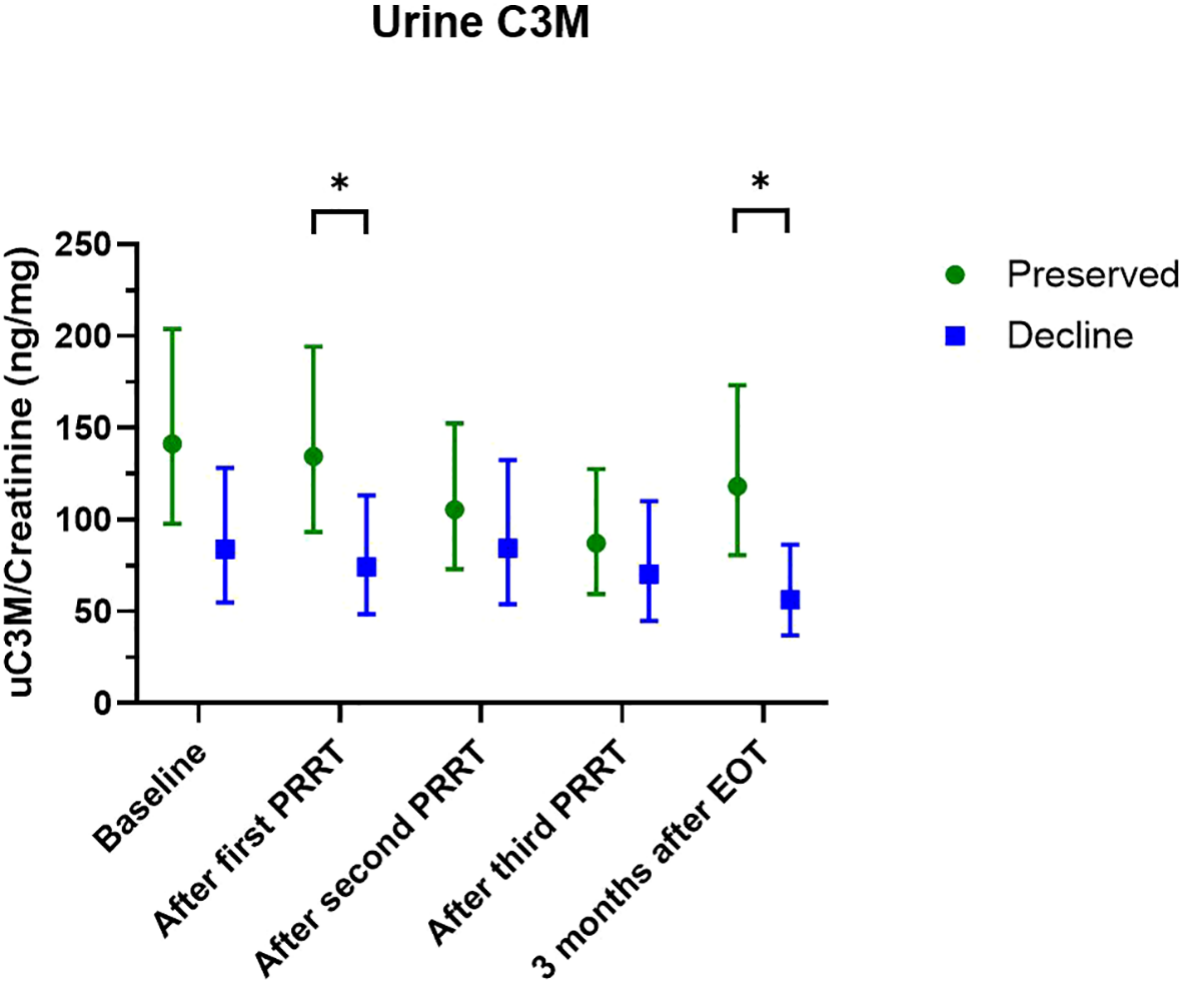
Levels of uC3M at each visit according to groups. Data are presented as estimated means and 95% confidence interval. Data are log-transformed and presented in this figure as back-transformed. Comparisons between groups are performed using linear mixed model. Green color: preserved kidney function-group, blue color: decline in kidney function-group. *P-value < 0.05.

When further testing for differences in uC3M at specific time points, we observed significantly lower levels of uC3M after the first PRRT treatment (74 ng/mg (95% CI: 49-113) *vs* 135 ng/mg (95% CI: 93-194), p=0.04) and three months after EOT (56 ng/mg (95% CI: 37-86) *vs* 118 (95% CI: 81-173) p=0.01) in the group of patients who subsequently declined in kidney function (**Figure 2**; **Supplementary Table 1**).

Raw data of all the fibrosis markers are presented in **Supplementary Table 2**, **Supplementary Figures 1, 2**.

### 3.4 Correlation between fibrosis markers and kidney function

We observed a significant correlation between sPRO-C6 and kidney function three months after EOT (spearman’s rho: -0.60, p: 0.03) (**Table 3**). For the remaining fibrosis markers, no statistically significant correlations to kidney function were observed, however uPRO-C6 at three months after EOT (spearman’s rho: -0.45, p: 0.13), and uC3M at baseline (spearman’s rho: 0.44, p: 0.12) had moderate correlations (**Table 3**).

**Table 3:** Spearman’s correlation between fibrosis markers and kidney function.

Fibrosis marker	Time point	Spearman’s rho	p-value
Serum PRO-C3	Baseline	0.37	0.20
	3 months follow up	0.09	0.76
Serum PRO-C6	Baseline	-0.16	0.58
	3 months follow up	-0.60	0.03
Serum C3M	Baseline	0.06	0.85
	3 months follow up	0.29	0.33
Urine PRO-C3	Baseline	-0.12	0.69
	3 months follow up	0.13	0.68
Urine PRO-C6	Baseline	0.35	0.21
	3 months follow up	-0.45	0.13
Urine C3M	Baseline	0.44	0.12
	3 months follow up	0.31	0.31

## 4 Discussion

In this prospective and exploratory cohort study, we investigated the potential of non-invasive fibrosis markers as prognostic markers for decline in kidney function in NEN-patients undergoing PRRT treatment. The overall levels of uC3M differed between patients who preserved and patients who declined in kidney function with significantly lower levels at the initiation of PRRT treatment and three months after EOT in patients who subsequently declined in kidney function, suggesting that uC3M holds the potential as a prognostic marker of future kidney function decline. However, these results are exploratory and larger studies are needed to draw firm conclusions about the usefulness.

Several previous prospective studies have investigated PRO-C6 and C3M as prognostic markers in different etiologies of CKD. Interestingly, most of these studies have confirmed an association between PRO-C6 and C3M with incidence of adverse kidney outcomes. *Genovese* et al. (17, 23) and *Pilemann-Lyberg* et al. (22) observed decreasing levels of uC3M with decreasing kidney function and an inverse association between uC3M and disease progression. However, after adjustment this association was non-significant in the latter study (22). Furthermore, *Genovese* et al. (17) demonstrated that baseline levels of uC3M were significantly lower in patients who subsequently had a decline in the kidney function (17). This is in line with our study where levels of uC3M after first treatment and after EOT were lower in the group of patients who subsequently experienced a decline in kidney function. This emphasizes the potential of uC3M to predict future kidney function decline.

*Sparding* et al. (21), *Pilemann-Lyberg* et al. (22), and *Møller* et al. (25) demonstrated that serum/plasma PRO-C6 (endotrophin) was independently associated with decline in kidney function in cohorts of patients with different etiology of kidney disease. *Neprasova* et al. (24) showed that PRO-C6 improved the prognostic ability of adverse renal outcomes when added to more common clinical variables. In our study, we were unable to demonstrate any overall differences in the levels of sPRO-C6 between patients who preserved and patients who declined in kidney function.

Results on urinary PRO-C6 are conflicting. One study showed an increased risk of kidney disease progression with higher levels of uPRO-C6 (20) whereas another study showed a protective effect of high uPRO-C6 levels (22). We were unable to demonstrate any association between uPRO-C6 and decline in kidney function.

In this study, the correlations between kidney function and three of the markers, sPRO-C6, uPRO-C6, and uC3M, were of moderate strength and they were comparable to previous studies (20, 21). This underscores the potential of the fibrosis markers as prognostic markers of decline in kidney function.

The lack of coherence between our findings and previous studies may be caused by the relatively small number of patients included in our study and hence a risk of type 2 error. Also, the pattern of changes in the fibrosis markers may depend on the etiology of the kidney disease, e.g. diabetic nephropathy, hypertension, IgA nephropathy etc. However, in larger studies of patients with CKD of several mixed etiologies both *Rasmussen* et al. (20) and *Genovese* et al. (23), demonstrated that the fibrosis markers were useful as prognostic markers of disease progression.

It is also important to consider the kidney function at the time of fibrosis marker measurement. In our study, the baseline GFR is 80 ml/min/1.73m^2^. This is considerable higher than the baseline GFR in most of the comparable studies. This may indicate that the fibrosis markers are more useful when some degree of kidney impairment is already present. This aligns with the results from *Pilemann-Lyberg* et al. (22) as the baseline mean eGFR was 82 ml/min/1.73m^2^ and they observed an independent association between sPRO-C6 and decline in eGFR for patients with baseline eGFR >30 and >45 ml/min/1.73m^2^. This association was insignificant for patients with eGFR >60 ml/min/1.73m^2^. In this study, no decline in kidney function was observed during the PRRT treatment. However, three months after the end of treatment, some renal impairment was observed, and at the end of follow up this impairment was even more pronounced. In contrast, lower levels of uC3M were observed already after the first PRRT treatment, which might indicate that changes in the fibrosis markers precedes the functional kidney impairment.

Little is known about the histopathological changes in the kidney following PRRT. There is a general understanding that chronic kidney disease is characterized by renal tubulointerstitial fibrosis regardless of the etiology (10–13). To our knowledge, only one case report has investigated the histopathology in patients with chronic kidney failure following PRRT (28). Kidney biopsies from three patients showed typical signs of thrombotic microangiopathy involving the glomeruli, arterioles, and small arteries, and, furthermore, tubular atrophy and interstitial fibrosis were dominant in all three cases. However, the number of kidney biopsies are too low to draw any firm conclusions about fibrosis.

The proportion of patients experiencing decline in kidney function following PRRT is remarkably high in this study (43%) compared with previous studies (8, 29). Even though not statistically significant, there appeared to be a higher proportion of patients with hypertension, prior PRRT treatments, and Yttrium-based treatments in the group of patients experiencing a decline in kidney function. Additionally, the baseline standard-GFR prior to PRRT tended to be lower in this group, although this was also not statistically significant. These are all factors that potentially contribute to a greater decrease in renal function following PRRT, and this may explain the high number of patients experiencing decline in kidney function following PRRT. The lack of statistical significance between the groups is likely to be attributable to the low number of patients.

A major strength of this study is the accurate measurement of the glomerular filtration rate using the ^51^Cr-EDTA or ^99^mTc-DTPA plasma clearance test instead of estimated glomerular filtration rate based on serum creatinine. However, the number of patients is low making the study vulnerable to type 2 errors. Six patients died during treatment and were not included in the final analysis due to missing follow up test. Due to the explorative design of the study, several statistical tests are performed which increases the risk of type 1 errors. Furthermore, our patient population is heterogenous encompassing all primary tumors including small intestinal NENs which are more likely to cause fibrotic complications (e.g. mesenteric fibrosis and carcinoid heart disease) than pancreatic and lung NENs. The contribution of fibrosis markers from other organs than the kidney could potentially interfere with the interpretation of the results. These limitations taken into consideration, our results should be interpreted cautiously and are meant as exploratory results that can serve as guidance when larger studies concerning fibrosis markers in patients with NENs are planned.

In conclusion, we investigated the potential of non-invasive fibrosis markers as prognostic markers of decline in kidney function in NEN-patients undergoing PRRT. After the first PRRT treatment and three months after EOT, we observed lower levels of uC3M in patients who subsequently experienced a decline in kidney function. These results suggest that uC3M holds the potential as a prognostic marker. However, the results should be considered exploratory, and one should be cautious in drawing firm conclusions about the usefulness. For this purpose, we suggest that larger prospective studies are carried out.

## Data Availability

The original contributions presented in the study are included in the article/**Supplementary Material**. Further inquiries can be directed to the corresponding author.
