# Supplementary material for: Fibrosis markers as prognostic markers of decline in kidney function in patients with neuroendocrine neoplasms undergoing peptide receptor radionuclide therapy
Source: Front Endocrinol (Lausanne). 2025 Jun 27;16:1495369. doi: 10.3389/fendo.2025.1495369 (PMC12245698; doi:10.3389/fendo.2025.1495369)

**Supplementary table 1.** Estimated means of fibrosis markers according to group at the different time points and relative difference between groups at all time points. Estimated means are estimated on log-transformed data and presented in this table as back-transformed.

| **Fibrosis**  **marker** | **Estimated**  **mean** | **95% CI** | **Estimated**  **mean** | **95% CI** | **Relative**  **difference** | **95% CI** | **P-value** |
| --- | --- | --- | --- | --- | --- | --- | --- |
|  | **Preserved** | | **Decline** | |  |  |  |
| **sPRO-C3** (ng/ml) | | | | | | | |
| Visit 1 | 12.6 | 9.3 – 17.0 | 11.7 | 8.3 – 16.6 | 0.93 | 0.59 – 1.48 |  |
| Visit 2 | 15.6 | 11.5 – 21.0 | 10.8 | 7.7 – 15.2 | 0.69 | 0.44 – 1.09 |  |
| Visit 3 | 13.0 | 9.6 – 17.5 | 14.1 | 9.8 – 20.2 | 1.08 | 0.68 – 1.73 |  |
| Visit 4 | 13.9 | 10.2 – 18.9 | 15.8 | 11.0 – 22.7 | 1.14 | 0.70 – 1.84 |  |
| Visit 5 | 14.1 | 10.4 – 19.0 | 13.3 | 9.2 – 19.1 | 0.94 | 0.59 – 1.51 |  |
| **sPRO-C6** (ng/ml) | | | | | | | |
| Visit 1 | 8.9 | 7.5 – 10.6 | 8.7 | 7.1 – 10.6 | 0.97 | 0.75 – 1.26 |  |
| Visit 2 | 9.9 | 8.4 – 11.7 | 9.1 | 7.5 – 11.1 | 0.92 | 0.71 – 1.20 |  |
| Visit 3 | 8.5 | 7.2 – 10.1 | 9.1 | 7.4 – 11.2 | 1.06 | 0.82 – 1.39 |  |
| Visit 4 | 9.0 | 7.5 – 10.7 | 10.3 | 8.4 – 12.6 | 1.14 | 0.88 – 1.49 |  |
| Visit 5 | 8.6 | 7.3 – 10.2 | 9.6 | 7.9 – 11.8 | 1.12 | 0.85 – 1.45 |  |
| **sC3M** (ng/ml) | | | | | | | |
| Visit 1 | 13.9 | 11.7 – 16.5 | 17.5 | 14.4 – 21.3 | 1.26 | 0.97 – 1.63 |  |
| Visit 2 | 14.0 | 11.8 – 16.6 | 16.7 | 13.8 – 20.4 | 1.20 | 0.92 – 1.55 |  |
| Visit 3 | 13.9 | 11.8 – 16.5 | 16.4 | 13.4 – 20.1 | 1.17 | 0.90 – 1.54 |  |
| Visit 4 | 13.6 | 11.5 – 16.2 | 16.2 | 13.2 – 19.8 | 1.19 | 0.91 – 1.55 |  |
| Visit 5 | 14.8 | 12.5 – 17.5 | 16.4 | 13.4 – 20.0 | 1.11 | 0.85 – 1.45 |  |
| **uPRO-C3** (ng/mg) | | | | | | | |
| Visit 1 | 28.4 | 15.5 – 51.9 | 10.9 | 5.4 – 22.0 | 0.39 | 0.15 – 0.97 |  |
| Visit 2 | 38.4 | 21.0 – 70.2 | 17.8 | 8.9 – 35.8 | 0.46 | 0.18 – 1.17 |  |
| Visit 3 | 26.9 | 14.7 – 49.2 | 19.3 | 9.4 – 39.8 | 0.72 | 0.28 – 1.84 |  |
| Visit 4 | 29.9 | 16.1 – 55.6 | 17.2 | 8.3 – 35.3 | 0.57 | 0.22 – 1.49 |  |
| Visit 5 | 29.9 | 16.1 – 55.6 | 12.9 | 6.4 – 25.9 | 0.43 | 0.17 – 1.09 |  |
| **uPRO-C6** (ng/mg) | | | | | | | |
| Visit 1 | 6.91 | 2.84 – 16.82 | 2.49 | 0.89 – 6.96 | 0.36 | 0.09 – 1.40 |  |
| Visit 2 | 7.17 | 2.95 – 17.44 | 2.65 | 0.95 – 7.41 | 0.37 | 0.10 – 1.45 |  |
| Visit 3 | 2.89 | 1.19 – 7.03 | 2.77 | 0.92 – 8.32 | 0.96 | 0.23 – 3.94 |  |
| Visit 4 | 4.55 | 1.79 – 11.58 | 2.92 | 0.97 – 8.78 | 0.64 | 0.15 – 2.72 |  |
| Visit 5 | 4.23 | 1.66 – 10.76 | 5.24 | 1.88 – 14.62 | 1.23 | 0.31 – 4.95 |  |
| **uC3M** (ng/mg) | | | | | | | |
| Visit 1 | 141.3 | 97.9 – 203.9 | 83.9 | 54.9 – 128.2 | 0.59 | 0.34 – 1.04 | 0.07 |
| Visit 2 | 134.5 | 93.2 – 194.2 | 74.2 | 48.5 – 113.3 | 0.55 | 0.31 – 0.97 | 0.04 |
| Visit 3 | 105.6 | 73.1 – 152.4 | 84.5 | 53.9 – 132.3 | 0.80 | 0.45 – 1.43 | 0.45 |
| Visit 4 | 87.1 | 59.5 – 127.6 | 70.4 | 44.9 – 110.2 | 0.81 | 0.45 – 1.46 | 0.48 |
| Visit 5 | 118.2 | 80.7 – 173.2 | 56.4 | 36.9 – 86.3 | 0.48 | 0.27 – 0.84 | 0.01 |

Data are presented as estimated means (95% confidence interval). NB: Differences in levels between groups are only tested for uC3M.

**Supplementary table 2.** Levels of fibrosis markers according to group at the different time points. Raw data.

| Fibrosis marker | Baseline  (visit 1) | | After first PRRT  (visit 2) | | After second PRRT  Visit 3) | | After third PRRT  (visit 4) | | 3 months after last PRRT (visit 5) | |
| --- | --- | --- | --- | --- | --- | --- | --- | --- | --- | --- |
|  | Preserved | Decline | Preserved | Decline | Preserved | Decline | Preserved | Decline | Preserved | Decline |
| sPRO-C3  (ng/ml) | 10.9  (9.1-13.8) | 9.7  (9.0-15.3) | 15.3  (11.3-21.0) | 11.5  (10.7-11.9) | 11.3  (10.4-16.9) | 11.4  (9.7-17.9) | 14.1  (10.2-19.1) | 13.4  (10.8-16.6) | 13.8  (11.3-16.9) | 12.0  (10.2-13.2) |
| sPRO-C6  (ng/ml) | 8.7  (7.7-9.6) | 9.0  (8.7-9.3) | 10.4  (8.0-12.1) | 9.9  (7.6-11.0) | 8.6  (7.2-10-1) | 9.7  (7.8-10.0) | 7.9  (7.5-10.4) | 10.2  (10.1-11.5) | 8.0  (7.4-9.5) | 9.7  (9.4-10.8) |
| sC3M  (ng/ml) | 15.4  (11.1-17.1) | 15.7  (14.2-24.8) | 13.4  (12.0-16.1) | 15.7  (13.4-19.9) | 15.1  (12.6-16.8) | 14.3  (14.3-18.5) | 14.5  (12.5-16.2) | 15.6  (13.8-19.0) | 15.0  (12.6-17.2) | 14.4  (14.4-17.9) |
| uPRO-C3  (ng/mg) | 28.5  (12.0-71.3) | 10.8  (4.7-21.8) | 41.4  (16.9-84.4) | 19.9  (10.3-22.5) | 23.5  (12.6-45.4) | 21.9  (18.4-26.1) | 23.4  (16.8-72.9) | 21.5  (15.9-28.1) | 20.0  (13.4-49.4) | 14.0  (9.3-27.6) |
| uPRO-C6  (ng/mg) | 9.5  (2.0-18.1) | 2.5  (1.6-3.5) | 7.9  (2.0-22.6) | 2.1  (2.0-3.4) | 1.7  (1.4-9.0) | 3.0  (2.2-4.8) | 2.6  (1.5-23.4) | 2.2  (1.6-5.31) | 2.0  (0.84-49.4) | 2.5  (2.2-4.8) |
| uC3M  (ng/mg) | 124  (103-193) | 80  (59-115.) | 111  (101-203) | 83  (69-102) | 101  (75-128) | 87  (79-93) | 100  (60-115) | 61  (56-66) | 92  (79-183) | 71  (26-88) |

Data are presented as median (IQR).

**Supplementary table 3.** Estimated glomerular filtration rate (eGFR) according to group at different time points.

|  | **Preserved**  **(n = 8)** | **Decline**  **(n = 6)** |
| --- | --- | --- |
| Visit 1 | 89 (67 – 90) | 67 (64 – 89) |
| Visit 2 | 89 (64 – 90) | 71 (66 – 72) |
| Visit 3 | 88 (68 – 90) | 69 (68 – 78) |
| Visit 4 | 90 (68 – 90) | 70 (63 – 82) |
| Visit 5 | 86 (67 – 90) | 57 (57 – 72) |

Data are expressed as median (IQR). eGFR is expressed as ml/min/1.73m^2^.

**Supplement figure 1a-f.** Levels of fibrosis markers for each patient at each visit. Raw data presented. Green color: Preserved kidney function group. Blue color: Decline in kidney function group. a: sPRO-C3, b: sPRO-C6, c: sC3M, d: uPRO-C3, e: uPRO-C6, f: uC3M.

**
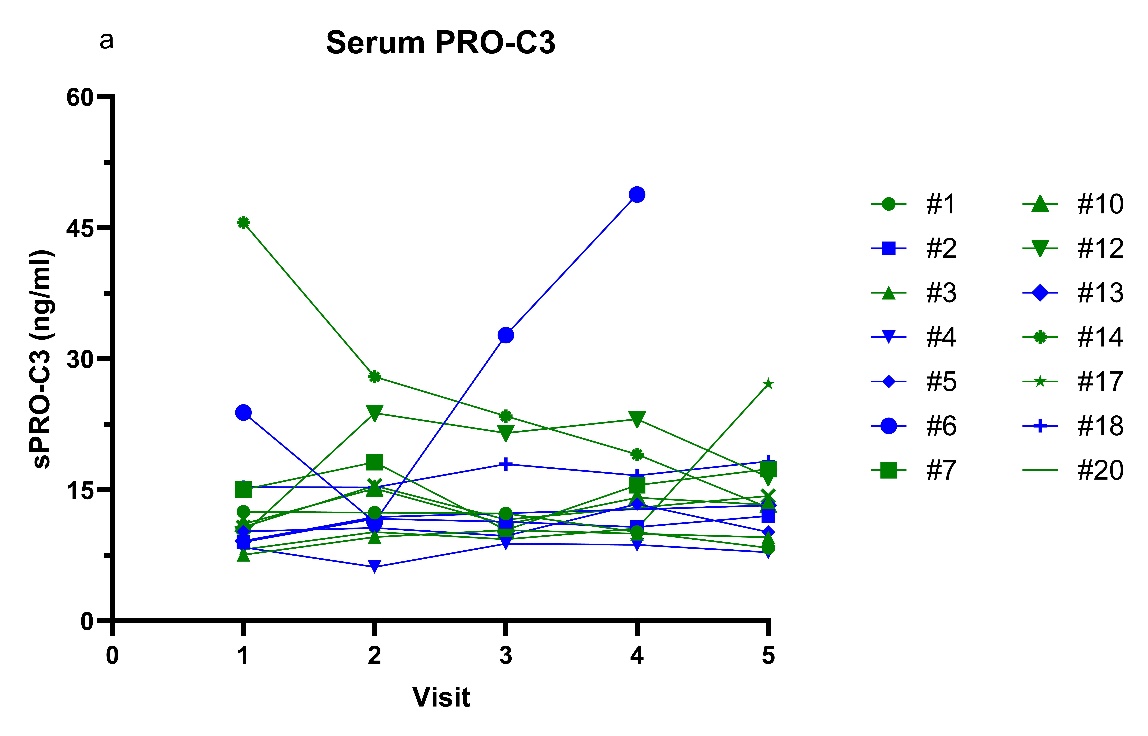
**

**
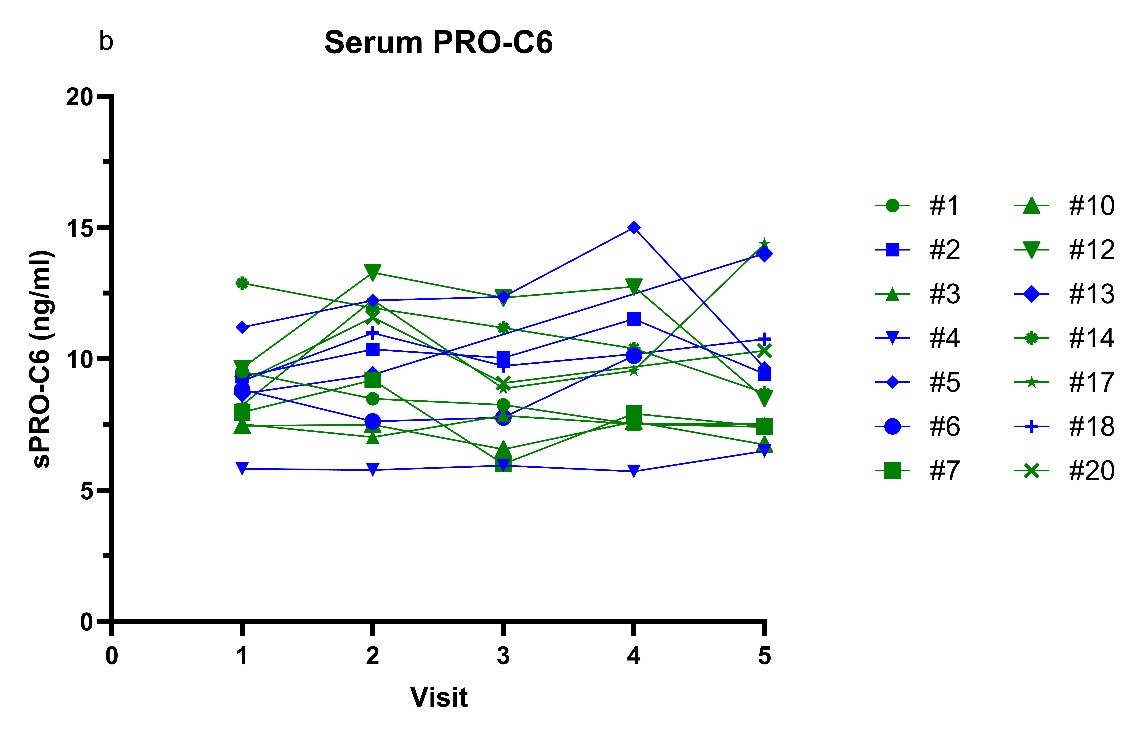
**

**
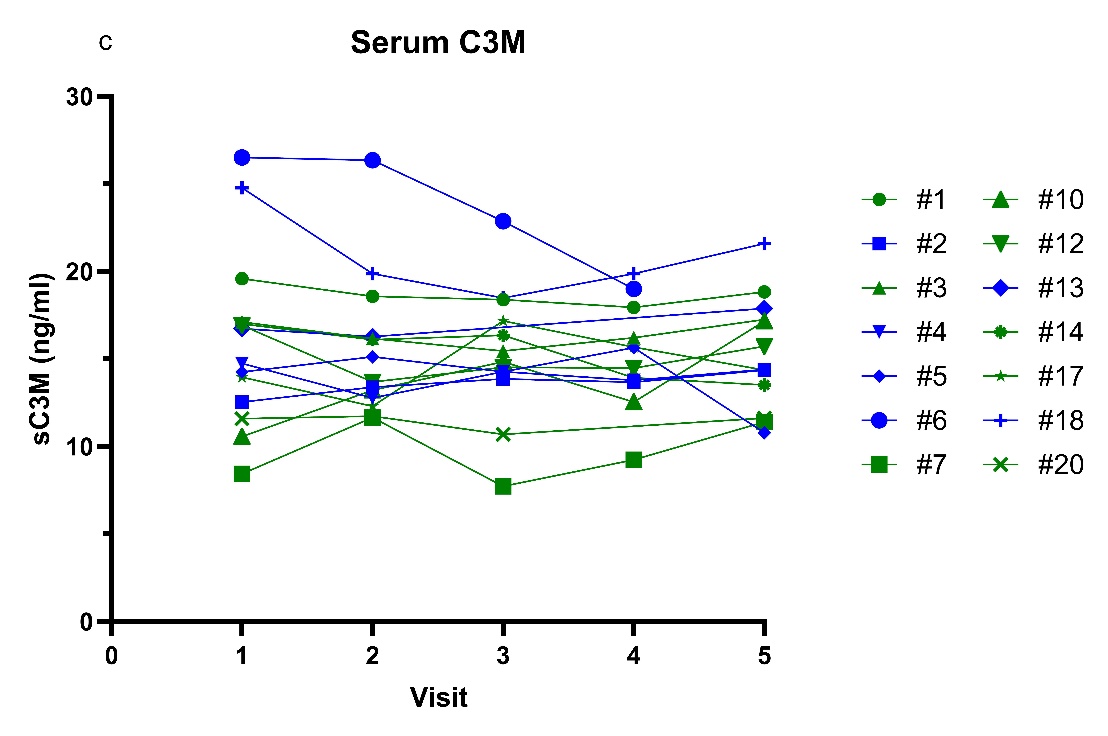
**


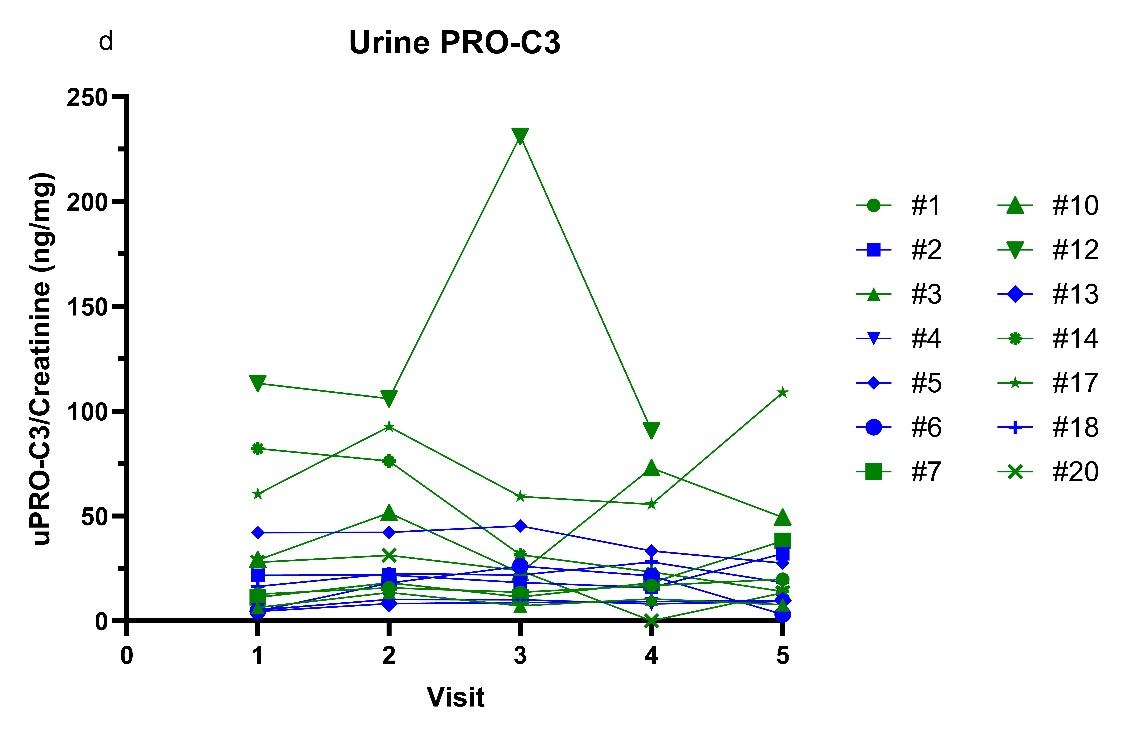


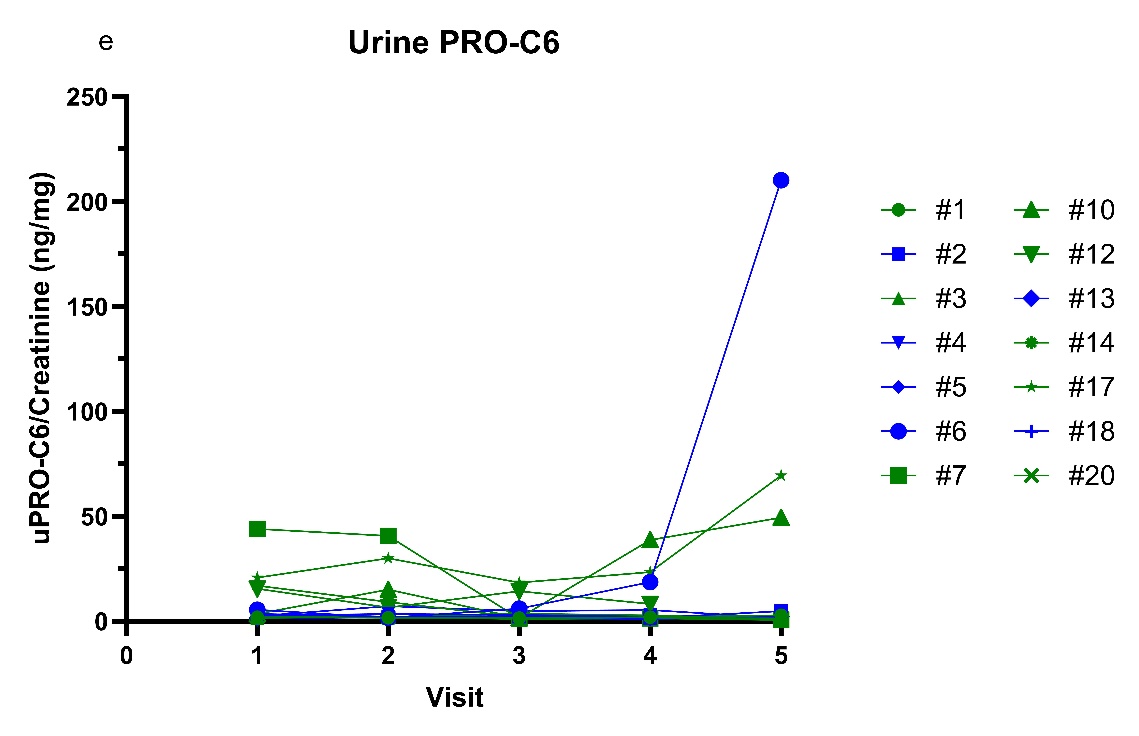


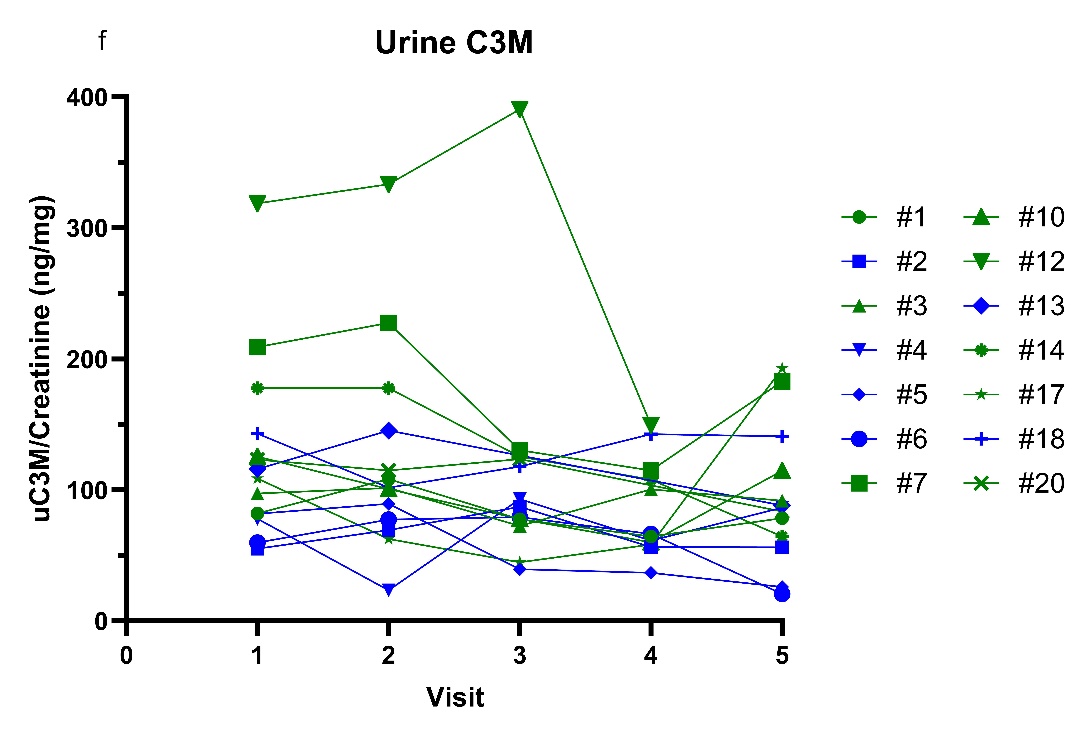


**Supplement figure 2a-f.** Levels of raw data fibrosis markers at each visit according to groups. Raw data are presented as median (IQR) and maximum and minimum value. a: sPRO-C3, b: sPRO-C6, c: sC3M, d: uPRO-C3, e: uPRO-C6, f: uC3M.


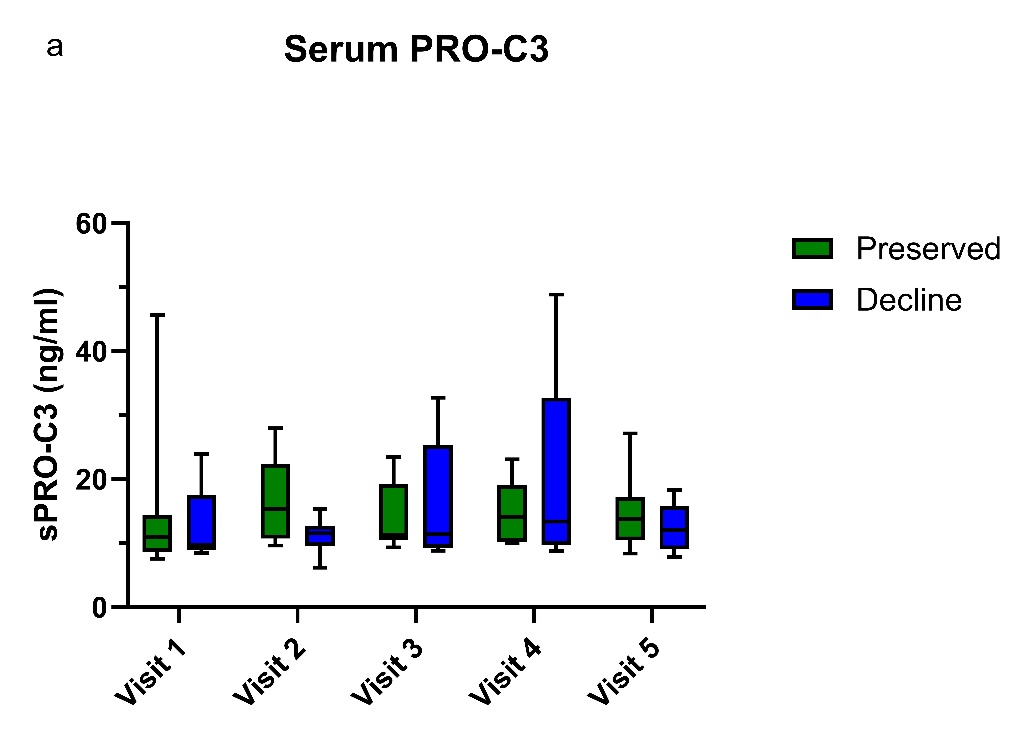


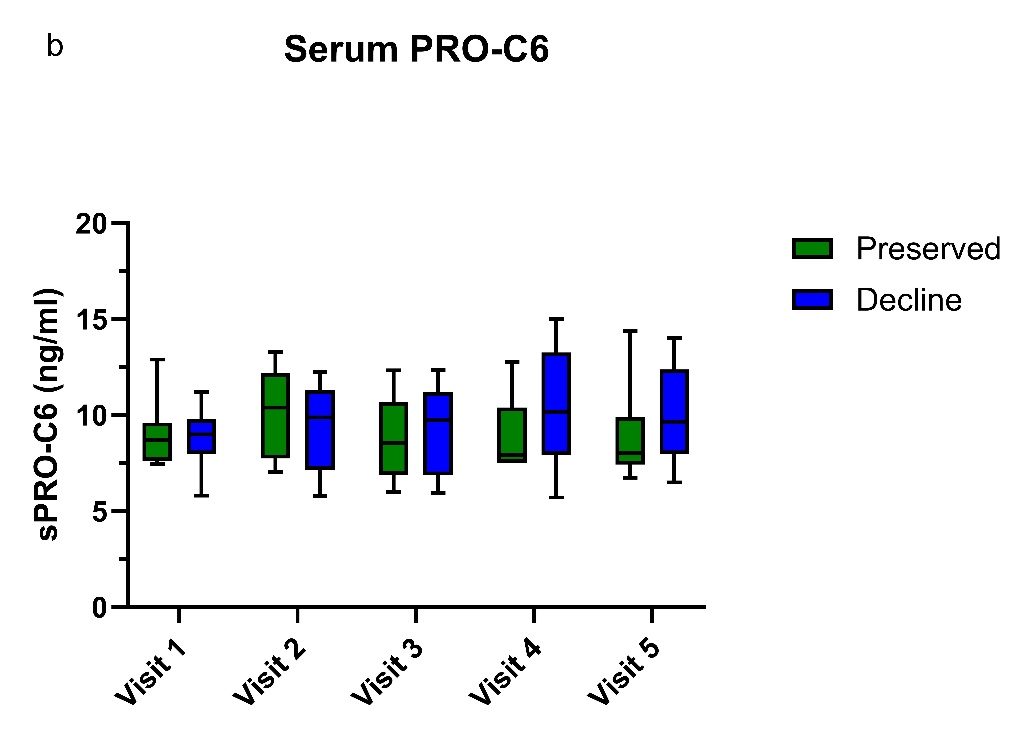


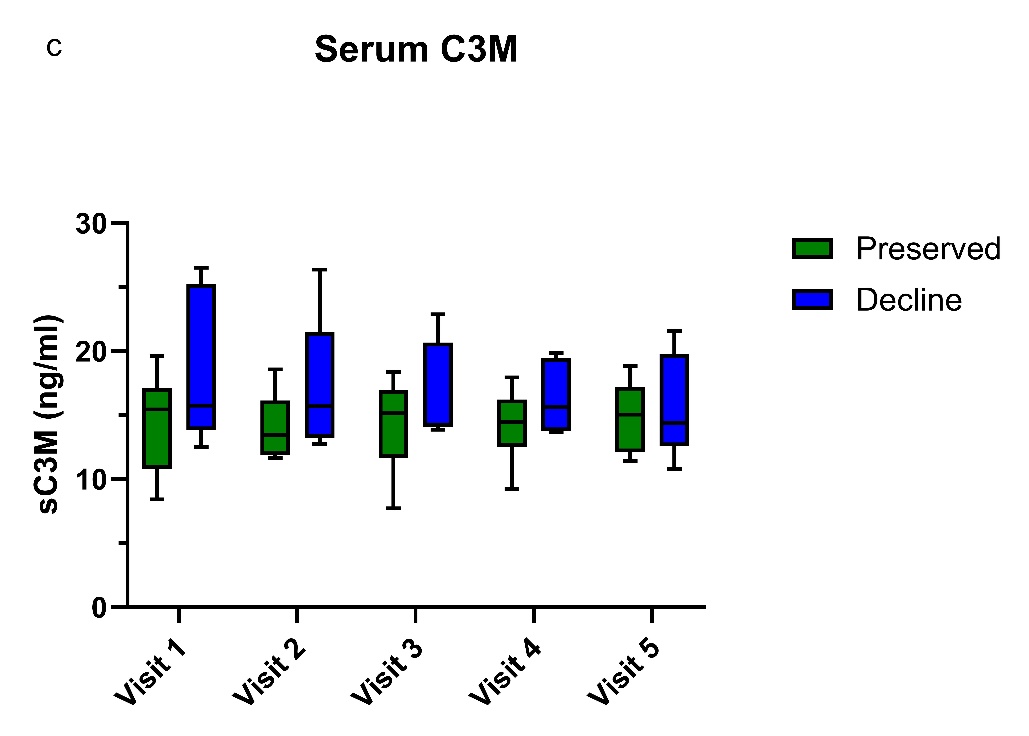


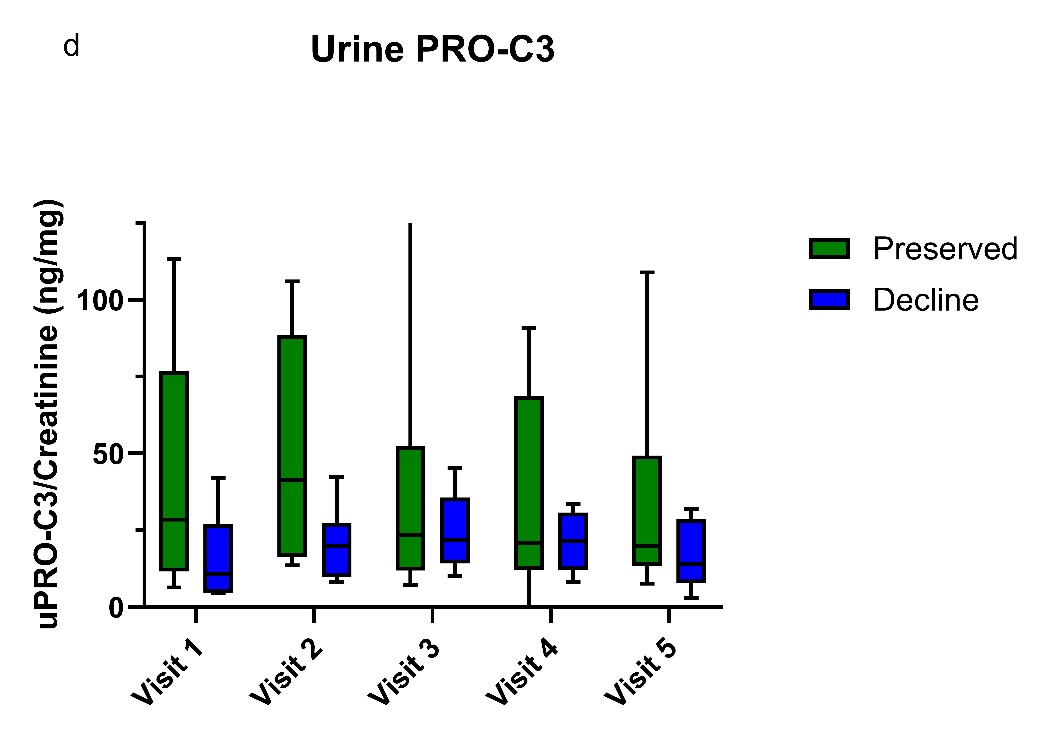


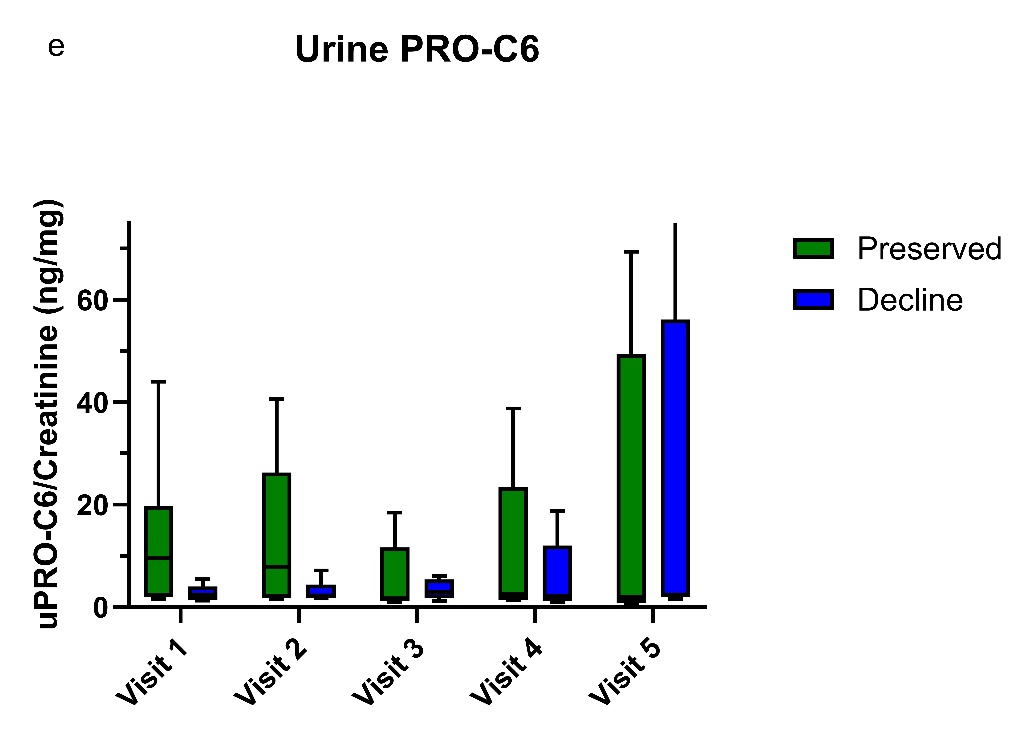


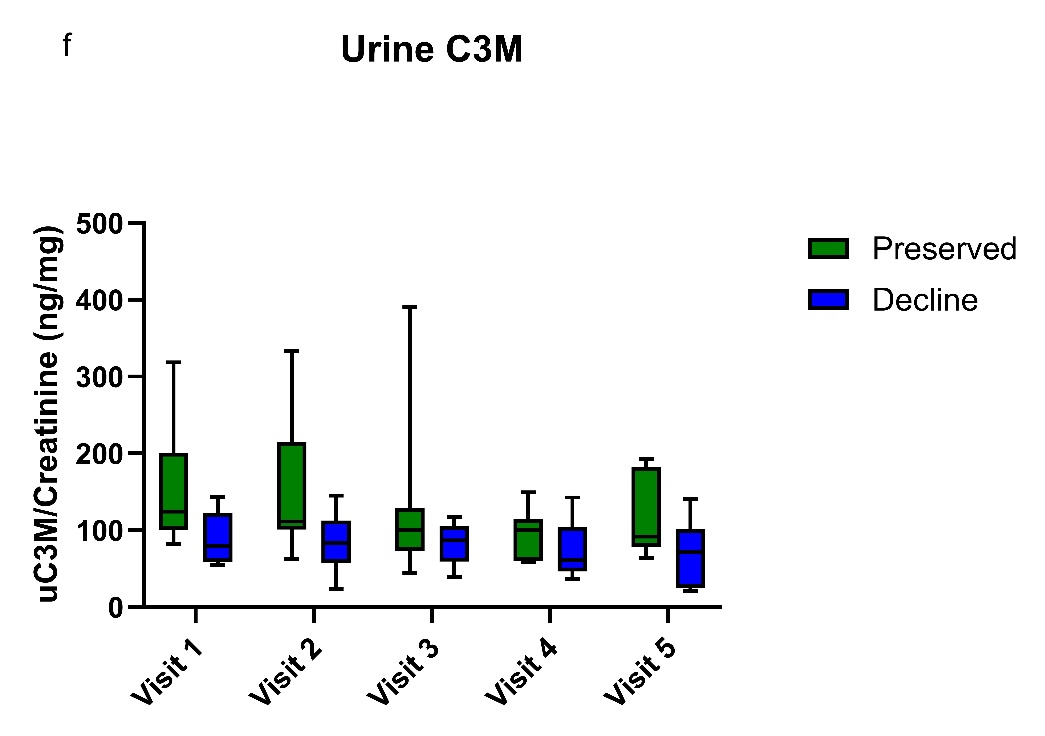

Supplement: Supplementary file 1 [file DataSheet1.docx]
